# P-LM421E8, the heparan sulfate chain-conjugated laminin-421-E8 fragment, drives differentiation of human induced pluripotent stem cells into hematopoietic progenitor cells comparable to basic fibroblast growth factor in a chemically defined system

**DOI:** 10.1016/j.mbplus.2025.100188

**Published:** 2025-12-10

**Authors:** Naoto Ninomiya, Kaoru Sasaki, Ryosuke Katori, Yasuhiro Shimizu, Kazumasa Fujita, Yukimasa Taniguchi, Taiko Kunieda, Kouichi Tamura, Masashi Yamada, Kiyotoshi Sekiguchi, Hironobu Kimura

**Affiliations:** a Research Division, Kobe Research Institute, HEALIOS K.K., Kobe, Hyogo, Japan; b Division of Matrixome Research and Application, Institute for Protein Research, The University of Osaka, Suita, Osaka, Japan

**Keywords:** Human induced pluripotent stem cells (hiPSCs), Hematopoietic progenitor cells (HPCs), Laminin E8 fragment, P-LM421E8, Basic fibroblast growth factor (bFGF), Natural killer cells, Chemically defined differentiation

## Abstract

Human induced pluripotent stem cells (hiPSCs) are a promising source for cell-based and regenerative therapies. In this study, we developed and optimized a chemically defined differentiation protocol to generate hematopoietic progenitor cells (HPCs) from hiPSCs. We demonstrated that basic fibroblast growth factor (bFGF) plays a crucial role in enhancing HPC differentiation, particularly when applied during both the mesoderm (ME) and hematopoietic endothelial (HE) induction stages. Additionally, we explored the use of P-LM421E8, a recombinant fusion protein of laminin421-E8 fragment with domain 1 (D1) of perlecan possessing heparan sulfate (HS) chains. Our findings indicate that P-LM421E8 effectively supports HPC differentiation, generating stable CD34-high/CD117^+^ populations comparable to those produced with bFGF treatment. Furthermore, we observed that HPCs generated on P‑LM421E8‑coated surfaces exhibited superior natural killer (NK) cell differentiation potential. Although both P-LM421E8 and bFGF supported HPC differentiation, no significant additive effects were observed when used together. This suggests that P-LM421E8 can effectively support HPC differentiation without the need for exogenous bFGF, possibly through enhanced interaction with endogenous FGFs. The structural properties of P-LM421E8, which facilitate retention and presentation of FGFs via HS chains on its D1, may contribute to its effectiveness in promoting HPC development. Our findings establish P‑LM421E8 as a potent matrix for HPC differentiation and highlight its promise for refining hematopoietic protocols and advancing cell‑based immunotherapies.

## Highlights

• P-LM421E8 promotes HPC differentiation from hiPSCs comparably to bFGF.
• hiPSCs cultured on P-LM421E8 yield HPCs with enhanced NK differentiation capacity.
• P-LM421E8 drives HPC differentiation without bFGF, likely via endogenous FGF signals.

## Introduction

Hematopoietic progenitor cells (HPCs) derived from induced pluripotent stem cells (iPSCs) have great promise for regenerative medicine and immunotherapy applications, particularly in the development of immune cell-based therapies [1], [2], [3]. The efficient generation of functional HPCs from iPSCs requires precise control over the differentiation process. This process typically involves the sequential transition from iPSCs to mesoderm, hemogenic endothelium, and finally, HPCs. However, optimizing culture conditions to improve the yield and quality of HPCs remains a significant challenge. To address these challenges, defined differentiation protocols have been developed, enabling the generation of HPCs under serum-free and feeder-free conditions using precise combinations of growth factors, small molecules, and extracellular matrix (ECM) components, thereby ensuring reproducibility, scalability, and medical applicability [4], [5], [6].

Fibroblast growth factor (FGF) signaling plays a pivotal role in mesoderm (ME) formation and the development of hematopoietic endothelial (HE) cells, functioning as a central regulator of early lineage commitment [7], [8], [9], [10], [11], [12]. This signaling pathway is intricately linked to Wnt and bone morphogenetic protein (BMP) signaling, both of which are essential for lineage specification [13], [14], [15]. FGF signaling promotes mesoderm induction by upregulating key transcription factors such as Brachyury and Tbx6, which are critical for gastrulation and the subsequent differentiation into mesodermal lineages [9], [16], [17]. In addition to its role in mesoderm development, FGF signaling acts in concert with vascular endothelial growth factor (VEGF) to drive the transition of mesodermal progenitors into HE cells by enhancing the expression of endothelial markers, including kinase insert domain receptor (KDR), also referred to as VEGF receptor 2, and CD34 [18], [19], [20].

Laminins are heterotrimeric glycoproteins composed of α, β, and γ chains, forming cross-shaped molecules that interact with cell surface receptors such as integrins and dystroglycan [21], [22], [23], [24], [25]. They are named according to their chain composition; for example, laminin-421 contains α4, β2, and γ1 chains. Among their functional domains, the E8 fragment (LM-E8) of laminins is a minimal binding domain that retains high-affinity interactions with integrins, regulating cell adhesion, migration, and differentiation similarly to full-length laminin [26], [27], [28], [29]. The application of recombinant LM-E8s in iPSC culture systems as xeno-free coating substrates has been shown to enhance maintenance and lineage-specific differentiation of human embryonic stem cells and iPSCs [30], [31], [32], [33], [34], [35]. Recently, our group introduced LM-E8s conjugated to perlecan domain 1 (D1) with heparan sulfate (HS) chains as a novel approach to improving iPSC differentiation [36], [37]. The modified LM-E8, particularly P-LM421E8 derived from laminin-421, has been shown to enhance FGF signaling by localizing bound FGFs closer to cell surface receptors, facilitating stronger pathway activation in concert with integrin-mediated pathways [36]. This coordinated signaling has been demonstrated to improve paraxial mesoderm formation and myogenic differentiation in human iPSC (hiPSC) cultures.

For differentiation of HPCs from hiPSCs, the protocol used in this study was built upon a previously established chemically defined system for inducing natural killer (NK) cells from human pluripotent stem cells [38]. Our results demonstrated that the addition of basic fibroblast growth factor (bFGF) during mesoderm and hemogenic endothelium induction significantly increased the proportion of HPCs with high CD34 and positive CD117 expression, indicating an improvement in HPC quality. Furthermore, we found that P-LM421E8 enhances HPC differentiation to an extent comparable to that achieved with bFGF supplementation. Moreover, HPCs generated on P-LM421E8-coated surfaces demonstrated enhanced potential to differentiate into NK cells. These results provide new insights into optimizing HPC differentiation strategies and may contribute to the advancement of cell-based immunotherapies by generating clinically relevant hematopoietic cell types.

## Results

### Effects of bFGF on HPC differentiation from hiPSCs

**Fig. 1:**
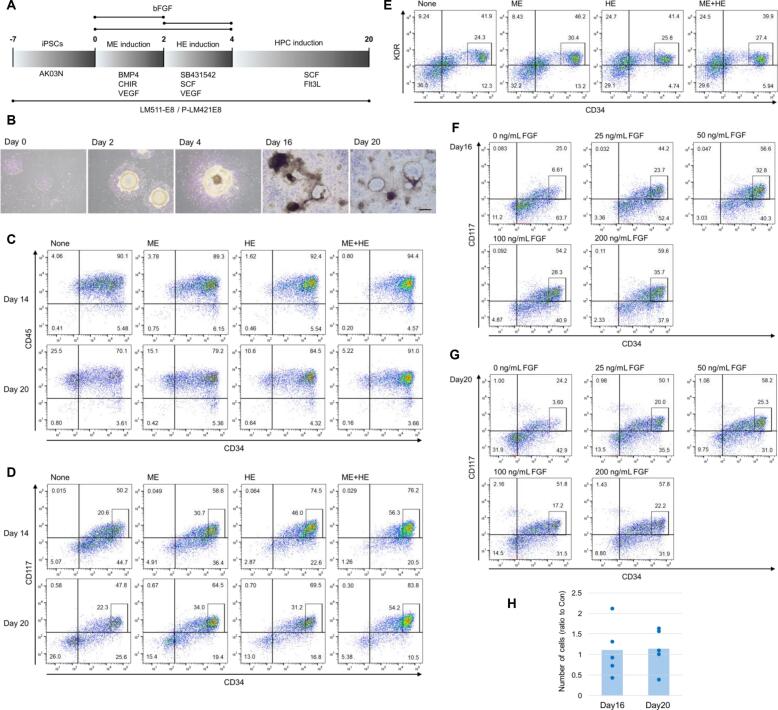
The effects of bFGF on HPC differentiation from hiPSCs. (A) Schematic representation of the differentiation process. hiPSCs were cultured at low density for 7 days before differentiation into HPCs through a sequential induction process: 2 days of ME induction, 2 days of HE induction, and 16 days of HPC differentiation. (B) Representative phase-contrast images at different stages of differentiation: iPSC culture (Day 0), ME induction (Day 2), HE induction (Day 4), and HPC induction (Day 16, 20). Scale bar: 500 µm. (C, D) Flow cytometry analysis of floating cells collected on days 14 and 20 of differentiation. bFGF was added during the ME induction period, HE induction period, or both (ME + HE). Cells were stained with anti-CD34 and anti-CD45 antibodies (C) or anti-CD34 and anti-CD117 antibodies (D). Boxed populations indicate CD34-high/CD117^+^ HPC cells. Representative plots from three independent differentiations are shown. (E) Flow cytometry analysis of adherent HE cells. After HE induction, cells were detached and stained with anti-CD34 and anti-KDR antibodies. Boxed populations represent CD34^+^/KDR^+^ HE cells. Representative plots from three independent differentiations are shown. (F, G) Dose-dependent effect of bFGF on HPC differentiation. bFGF was added at concentrations of 0, 25, 50, 100, or 200 ng/mL during both the ME and HE induction periods. Floating cells were collected on days 16 (F) and 20 (G) and analyzed by flow cytometry using anti-CD34 and anti-CD117 antibodies. Boxed populations indicate CD34-high/CD117^+^ HPC cells. Representative plots from two independent differentiations are shown. (H) Quantification of floating cell numbers. Cells were cultured with or without 50 ng/mL bFGF during the ME and HE induction periods. Floating cells were collected on days 16 and 20, and their numbers were quantified. Data represent the ratio of cell numbers in the presence versus absence of bFGF (Con: control) from five independent differentiation experiments.

We employed a chemically defined differentiation protocol to generate HPCs from hiPSCs, based on the method reported by Matsubara et al [38]. hiPSCs were seeded at low density on laminin-511-E8 fragment (LM511E8)-coated surfaces and cultured for seven days to form colonies (Fig. 1A, B). Following this, we initiated the differentiation process, which involved three key stages: ME induction using BMP4, a Wnt signaling activator, and VEGF; HE cell induction with a transforming growth factor-β (TGF-β) signaling inhibitor, stem cell factor (SCF), and VEGF; and HPC induction with SCF and Fms-related tyrosine kinase 3 ligand (Flt3L). During differentiation, hiPSC colonies underwent significant morphological changes, ultimately generating HPCs as floating cells derived from adherent cell structures (Fig. 1B).

To optimize differentiation efficiency, we investigated the effects of bFGF, given its previously reported role in promoting the induction of ME and HE cells [11], [12], [13], [14], [15], [16], [17], [18], [19]. We added bFGF during the ME induction period, the HE induction period, or both (Fig. 1A), and assessed its impact by analyzing the expression of CD34, CD117, and CD45 as HPC markers in floating cells collected on days 14 and 20 of differentiation. Our results demonstrated that the addition of bFGF during either the ME or HE induction period significantly increased the population of cells with high CD34 and positive CD117 expression (Fig. 1C, D), indicating that bFGF promotes the generation of functionally competent HPCs. Moreover, the simultaneous addition of bFGF during both the ME and HE periods further enhanced the production of CD34-high/CD117^+^ HPCs compared to its addition during a single period. These findings suggest that bFGF exerts an additive effect when applied at both induction stages, thereby improving the efficiency and quality of HPC differentiation from hiPSCs.

To investigate the underlying mechanism of this bFGF effect, we examined HE cells that were double-positive for KDR and CD34 on day 4 of differentiation. Our analysis revealed no significant difference in the proportion of KDR^+^/CD34^+^ HE cells among the non-treated control group and the bFGF-treated groups in which bFGF was added during the ME induction period, the HE induction period, or both periods (Fig. 1E).

Next, we investigated the concentration dependence of bFGF, testing doses of 0, 25, 50, 100, and 200 ng/mL during both the ME and HE induction periods. Our results showed that even at a concentration of 25 ng/mL, the effect of bFGF on HPC production was sufficient and not significantly enhanced by increase in concentration (Fig. 1F and G).

We also counted the number of floating cells produced on days 16 and 20. The results indicated that bFGF treatment did not significantly increase the overall production of floating cells, with mean (± SD) ratios relative to the no-bFGF condition of 1.10 ± 0.65 on day 16 and 1.14 ± 0.50 on day 20, pooled across five independent differentiations (Fig. 1H). However, pooled quantitative analyses of CD34‑high/CD117^+^ cells across five independent differentiations demonstrated a consistent bFGF effect, with mean (± SD) ratios relative to the no-bFGF condition of 2.94 ± 1.62 on day 16 and 2.73 ± 0.86 on day 20 (Fig. S1A). These findings suggest that bFGF does not enhance overall hematopoietic cell production but instead promotes the generation of stable, high-quality HPCs.

### Effects of P-LM421E8 on HPC differentiation from hiPSCs

**Fig. 2:**
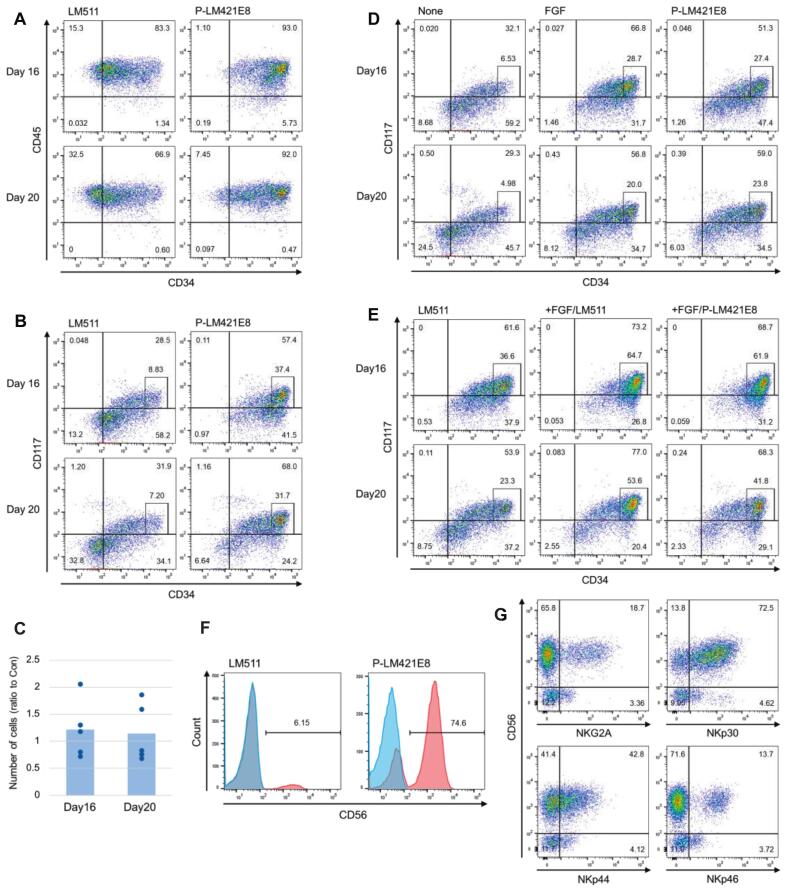
Effects of P-LM421E8 on HPC differentiation from hiPSCs. (A, B) Flow cytometry analysis of floating cells differentiated on LM511E8- or P-LM421E8-coated dishes. hiPSCs were seeded on LM511E8- or P-LM421E8-coated dishes and cultured for 7 days, followed by differentiation into HPCs through ME and HE induction. Floating cells collected on days 16 and 20 were stained with anti-CD34 and anti-CD45 (A) or anti-CD34 and anti-CD117 antibodies (B). Boxed populations represent CD34-high/CD117^+^ HPC cells. Representative plots from three independent differentiations are shown. (C) Quantification of floating cell numbers. hiPSCs were seeded on LM511E8- or P-LM421E8-coated dishes and differentiated into HPCs. Floating cells were collected on days 16 and 20, and their numbers were counted. Data represent the ratio of cell numbers in P-LM421E8-coated versus LM511E8-coated dishes (Con: control) from five independent differentiation experiments. (D) Flow cytometry analysis comparing the effects of P-LM421E8 and bFGF on HPC differentiation. hiPSCs were seeded on LM511E8 and differentiated without bFGF (None), with 50 ng/mL bFGF during the ME and HE induction periods (FGF), or on P-LM421E8 without bFGF (P-LM421E8). Floating cells collected on days 16 and 20 were analyzed using anti-CD34 and anti-CD117 antibodies. Representative plots from three independent differentiations are shown. (E) Flow cytometry analysis assessing potential additive effects of P-LM421E8 and bFGF on HPC differentiation. hiPSCs were differentiated in the presence of LM511E8 without bFGF (LM511), LM511E8 with 50 ng/mL bFGF during ME and HE induction (+FGF/LM511), or P-LM421E8 with bFGF (+FGF/P-LM421E8). Floating cells collected on days 16 and 20 were analyzed using anti-CD34 and anti-CD117 antibodies. Representative plots from three independent differentiations are shown. (F) NK cell differentiation from HPCs. HPCs obtained from hiPSCs seeded on LM511E8-coated or P-LM421E8-coated dishes were cultured under NK cell differentiation conditions for 42 days. Flow cytometry was performed using anti-CD56 antibodies. Blue and red histograms represent isotype control and anti-CD56 staining, respectively. Representative plots from two independent differentiations are shown. (G) Characterization of NK cells differentiated from hiPSC-derived HPCs on P-LM421E8. Flow cytometry analysis was performed using anti-CD56 in combination with anti-NKG2A, anti-NKp30, anti-NKp44, or anti-NKp46 antibodies. Representative plots from two independent differentiations are shown.

We previously developed P-LM421E8, a recombinant form of the D1 of perlecan conjugated to the C-terminus of LM421-E8, which facilitates bFGF signaling through its binding to bFGF via heparan sulfate chains on the D1, thereby promoting paraxial mesoderm formation and subsequent myogenic differentiation [36]. Given these properties, we examined the effects of P-LM421E8 on the differentiation of HPCs from iPSCs. iPSCs were plated on dishes coated with either LM511E8 or P-LM421E8, followed by differentiation into HPCs (Fig. 1A). On days 16 and 20, flow cytometric analysis using anti-CD34, anti-CD117, and anti-CD45 antibodies (Fig. 2A, B) revealed that the floating cells obtained from differentiation on P-LM421E8-coated surfaces exhibited higher and more stable expression of CD34 and CD117 compared to those obtained from differentiation on LM511E8-coated surfaces. Consistently, pooled quantitative analyses of CD34‑high/CD117^+^ cells across five independent differentiations showed a clear effect of P‑LM421E8, with mean (± SD) ratios relative to LM511E8 of 3.19 ± 1.30 on day 16 and 3.38 ± 1.45 on day 20 (Fig. S1B). These results strongly indicate that P-LM421E8 coating is highly effective in producing high-quality HPCs compared with LM511E8 coating. The total number of floating cells was slightly higher in the P-LM421E8-coated condition, although the difference was not statistically significant, with mean (± SD) ratios relative to LM511E8 of 1.21 ± 0.53 on day 16 and 1.14 ± 0.54 on day 20, pooled across five independent differentiations (Fig. 2C). This result closely resembled those from experiments evaluating the effects of bFGF.

To assess substrate effects attributable to differing laminin α chains and the specific function of the perlecan D1 module, we performed direct comparative differentiations using LM511E8, LM421E8, and P‑LM421E8. In two independent differentiation experiments (Fig. S2), P‑LM421E8 consistently yielded higher fractions of CD34‑high/CD117^+^ cells than LM511E8 and LM421E8 on both days 16 and 20. By contrast, LM421E8 and LM511E8 produced broadly comparable HPC outputs, with LM511E8 showing a slight tendency to yield higher fractions. These findings support the notion that the perlecan D1 module in P‑LM421E8 contributes to enhancing HPC generation beyond the activity of the core laminin E8 fragments.

We next compared the effects of P-LM421E8 and bFGF directly. As shown in Fig. 2D, P-LM421E8 alone achieved a level of HPC production (CD34‑high/CD117^+^) similar to that observed with bFGF. To assess whether P-LM421E8 and bFGF exert additive effects, we compared three conditions: LM511E8 coating alone, LM511E8 coating with bFGF addition, and P-LM421E8 coating with bFGF addition (Fig. 2E). Our results showed that the combination of P-LM421E8 and bFGF did not result in a significant additive effect. These findings suggest that P-LM421E8 effectively supports HPC differentiation in a manner comparable to bFGF treatment.

Given that NK cell differentiation from iPSC-derived HPCs has been demonstrated in the system we employed [38], we assessed the functional capacity of HPCs differentiated on P-LM421E8 to generate NK cells. HPCs obtained under P-LM421E8-coated and LM511E8-coated conditions were subjected to NK differentiation by culturing in the presence of IL-15, IL-7, SCF, and Flt3L. On day 42, more than 70 % of cells derived from HPCs generated on P‑LM421E8 were CD56‑positive, whereas only about 6 % of those derived from HPCs generated on LM511E8 expressed CD56 (Fig. 2F). Furthermore, CD56-positive cells differentiated from HPCs produced on P-LM421E8 included populations expressing NKG2A, NKp30, NKp44, and NKp46, indicative of functional NK cells (Fig. 2G). In another experiment, LM511E8-derived HPCs achieved > 35 % NK differentiation, although the efficiency remained well below that of P‑LM421E8-derived HPCs (>90 %; Fig. S3). Furthermore, phenotypic analysis indicated that the resulting NK cells were less mature than those derived from P‑LM421E8, with lower expression of NKp30/NKp44/NKp46 (especially NKp44) and higher expression of NKG2A (Fig. S3). Together, these results indicate that HPCs generated on P‑LM421E8, compared with those generated on LM511E8, possess a superior ability to undergo NK differentiation and yield more mature NK cells, demonstrating their higher functional quality.

## Discussion

In this study, we optimized the differentiation of HPCs from hiPSCs using a chemically defined protocol. Our findings revealed that bFGF enhances HPC differentiation, particularly when applied during both the ME and HE induction stages. Furthermore, we investigated the role of P-LM421E8, a recombinant fusion protein composed of LM421-E8 and the D1 domain of perlecan and we demonstrated comparable support for HPC differentiation provided by P-LM421E8 to that provided by bFGF. Remarkably, HPCs generated on P-LM421E8-coated surfaces showed superior differentiation capacity into NK cells compared to those obtained from cultures on surfaces coated with LM511E8, which has been previously used as a substrate.

The simultaneous application of P-LM421E8 and bFGF did not further enhance HPC differentiation, indicating that P-LM421E8 alone is sufficient to support hematopoietic differentiation at levels comparable to those achieved with bFGF. This finding is consistent with our previous work, which demonstrated that exogenous bFGF does not augment the effect of P-LM421E8 on paraxial mesoderm differentiation, where P-LM421E8 acts through the amplification of endogenous FGF signaling [36]. Thus, the absence of an additive effect in the present study suggests that P-LM421E8 similarly enhances HPC differentiation by amplifying endogenous FGF signaling. Given that the HS chains on the D1 domain of perlecan facilitate the binding of FGF to its receptor (FGFR) and promote FGFR dimerization [39], [40], [41], it is likely that P-LM421E8 recruits and concentrates endogenous FGF at the basal surface of adherent cells, thereby enhancing localized FGF signaling.

Treatment with bFGF during the ME and HE induction stages did not increase the population of CD34^+^/KDR^+^ HE cells; however, it significantly enhanced the proportion of HPCs with high CD34 and positive CD117 expression. This suggests that, contrary to previous reports [18], [19], bFGF does not promote the induction or enhance the stability of HE cells but rather improves their competency for subsequent HPC differentiation. Another notable observation was that, while the total number of floating hematopoietic cells remained largely unchanged following bFGF treatment, the proportion of cells with high CD34 and positive CD117 expression increased remarkably. A similar trend was observed in our differentiation experiments using P-LM421E8, indicating that both P-LM421E8 and bFGF primarily improve the quality and stability of HPCs rather than the quantity of HPCs derived from HE cells.

In our HPC differentiation system, both floating HPCs and adherent cells were present. The adherent cell population included CD73^+^ mesenchymal cells and CD31^+^ endothelial cells (data not shown), suggesting that P-LM421E8 and bFGF may influence these cell populations. Given the potential role of these adherent cells in forming a niche for HPCs [42], [43], [44], [45], they may contribute to the maintenance and modulation of HPCs during differentiation. These observations point to dynamic interactions between HPCs and their microenvironment that could affect HPC stability over time. Thus, P-LM421E8 and bFGF may enhance the quality and stability of HPCs by modulating the differentiation and behavior of adherent cells derived from hiPSCs.

We noted variability in the absolute percentages of CD34‑high/CD117^+^ cells across independent differentiations, even when the overall protocol was kept the same. This is likely because hiPSC differentiation at low seeding density is sensitive to several practical parameters, including variation in iPSC passage number, subtle differences in the number of colonies at induction, the timing of medium changes and cytokine additions, and minor operator‑dependent handling (e.g., pipetting‑induced shear that can inadvertently detach adherent colonies). Such factors can contribute to run‑to‑run differences in absolute readouts. While our conclusions are supported by pooled quantitative analyses across independent experiments (Fig. S2), we recognize the need to further reduce variability. To that end, we plan to implement measures to improve robustness, including maintaining more consistent seeding densities, defining a clear optimal iPSC passage range, harmonizing media-change schedules, and adopting standardized, step‑by‑step handling procedures. We anticipate that these steps will help stabilize outcomes and facilitate broader reproducibility.

In conclusion, this study establishes P-LM421E8 as a potent substrate for HPC differentiation, comparable to bFGF in promoting hematopoietic lineage commitment. The enhanced NK cell differentiation observed in HPCs produced with P-LM421E8 highlights its potential application in NK cell-based immunotherapies. Further investigations into the mechanisms underlying P-LM421E8- and bFGF-mediated differentiation could provide valuable insights into its therapeutic potential for generating hematopoietic and immune cells from iPSCs, ultimately advancing their therapeutic applications for generating clinically relevant cell populations from iPSCs.

## Materials and methods

### Recombinant laminin fragment proteins

The recombinant laminin fragment P-LM421E8 was produced using the Freedom^TM^ CHO-S^TM^ Kit (Life Technologies). Briefly, cDNAs encoding the α4-E8 fragment C-terminally conjugated to the D1 of perlecan, β2-E8, and γ1-E8 [36] were introduced into the pCHO 1.0 expression vector (Life Technologies), followed by selection of CHO cell clones stably expressing recombinant P-LM421E8 through a series of limiting dilutions. Recombinant P-LM421E8 secreted by the CHO cells was purified by anion exchange chromatography using HiTrap Q HP (Cytiva) and following hydrophobic interaction chromatography using HiTrap Phenyl HP. The purified P-LM421E8 was dialyzed against PBS (−) at pH 7.4. Protein concentrations were determined using a BCA protein assay kit (Thermo Fisher Scientific), with bovine serum albumin as the standard. Authenticity of the purified P-LM421E8 was confirmed by SDS-PAGE. iMatrix-511 (Matrixome/Nippi) was used as the LM511E8 substrate. Coating of P-LM421E8 and LM511E8 was performed at 1.0 µg/cm^2^ and 0.5 µg/cm^2^, respectively, in PBS (−) at pH 7.4.

### Culture of hiPSCs

The hiPSC line used in this study was derived from umbilical cord blood CD34^+^ cells according to previously established protocols [46]. The cells were generated at the Lonza Walkersville facility (Lonza Laboratories) and maintained in AK03N medium (Ajinomoto) on iMatrix-511-coated dishes. Cultures were incubated at 37℃ in a humidified atmosphere containing 5 % CO_2_.

### Differentiation of HPCs

The differentiation of hiPSCs into HPCs was conducted based on a previously established protocol [38]. hiPSCs were seeded at a density of 3.0 × 10^3^ cells per 60-mm dish coated with iMatrix-511 or P-LM421E8 and cultured in AK03N medium supplemented with 10 µM Y-27632 (FUJIFILM Wako Pure Chemical). Two days after seeding, the medium was replaced with AK03N without Y-27632, and the culture was continued for an additional five days. For ME induction, the medium was changed to DMEM/F12 (Thermo Fisher Scientific) containing 80 ng/mL BMP4 (FUJIFILM Wako Pure Chemical), 2 µM CHIR99021 (FUJIFILM Wako Pure Chemical), and 80 ng/mL VEGF165 (FUJIFILM Wako Pure Chemical). Cells were cultured in this medium for two days. Following ME induction, HE cell differentiation was induced by replacing the medium with Essential 6 (Thermo Fisher Scientific) supplemented with 80 ng/mL VEGF165, 50 ng/mL SCF (FUJIFILM Wako Pure Chemical), and 2 µM SB431542 (FUJIFILM Wako Pure Chemical). Cells were maintained in this medium for another two days. To promote HPC differentiation, the culture medium was changed to StemPro-34 SFM (Thermo Fisher Scientific) supplemented with 50 ng/mL SCF and 50 ng/mL FLT3L (PeproTech), and the differentiation was continued for 10 to 16 days. The effects of bFGF were evaluated by adding bFGF (FUJIFILM Wako Pure Chemical) during either the ME or HE induction period, or both. Floating cells were harvested at defined time points during differentiation and analyzed using a FACSVerse flow cytometer (BD Biosciences) with anti-CD45 (clone HI30; BD Biosciences), anti-CD34 (clone 8G12; BD Biosciences), and anti-CD117 (clone 104D2; BD Biosciences) antibodies. For the assessment of HE cells, adherent cells were detached using TrypLE Select (Thermo Fisher Scientific) following HE induction and analyzed by flow cytometry using anti-KDR (clone 89106; BD Biosciences) and anti-CD34 (clone 8G12; BD Biosciences) antibodies.

### Differentiation of NK cells

For NK cell differentiation, HPCs were cultured in CTS AIM V medium (Thermo Fisher Scientific) supplemented with 5 % (v/v) heat-inactivated fetal bovine serum (Sigma Aldrich), 100 µg/mL SCF, 100 µg/mL FLT3L, 50 µg/mL IL-7 (PeproTech), and 50 µg/mL IL-15 (PeproTech). The medium was changed every three to four days by replacing half of the volume. NK cell differentiation was evaluated by flow cytometry using anti-CD56 (clone B159; BD Biosciences), anti-NKG2A (clone REA110; Miltenyi Biotec), anti-NKp30 (clone P30-15; BioLegend), anti-NKp44 (clone P44-8; BioLegend), and anti-NKp46 (clone 9E2; BioLegend) antibodies.

## CRediT authorship contribution statement

**Naoto Ninomiya:** Writing – review & editing, Methodology, Investigation, Data curation, Conceptualization. **Kaoru Sasaki:** Writing – review & editing, Methodology, Investigation, Data curation, Conceptualization. **Ryosuke Katori:** Writing – review & editing, Methodology, Investigation, Data curation, Conceptualization. **Yasuhiro Shimizu:** Writing – review & editing, Resources, Investigation. **Kazumasa Fujita:** Writing – review & editing, Resources, Investigation. **Yukimasa Taniguchi:** Writing – review & editing, Resources, Investigation. **Taiko Kunieda:** Writing – review & editing, Resources, Investigation. **Kouichi Tamura:** Writing – review & editing, Supervision, Project administration. **Masashi Yamada:** Writing – original draft, Visualization, Supervision, Methodology, Formal analysis, Conceptualization. **Kiyotoshi Sekiguchi:** Writing – review & editing, Supervision, Resources, Project administration, Funding acquisition, Conceptualization. **Hironobu Kimura:** Writing – review & editing, Supervision, Project administration, Conceptualization.

## Funding

The studies done by N.N., K.S., R.K., K.T., M.Y. and H.K. were funded by HEALIOS K.K. K.S. was supported by grants from the Japan Agency for Medical Research and Development (AMED) JP17bm0404005h and JP20bm0804025 for production of P-LM421E8 using CHO-S cells. K.S. is supported by a KAKENHI for Transformative Research Area (A) 23,721,401 from the Ministry of Education, Culture, Sports, Science, and Technology (MEXT) of Japan.

## Declaration of competing interest

The authors declare the following financial interests/personal relationships which may be considered as potential competing interests: H.K. is Executive Officer of HEALIOS K.K. N.N., K.S., R.K., K.T., and M.Y. are employees of HEALIOS K.K. Y.S., Y.T., and T.K. are employees of Matrixome, Inc. K.F. is an employee of Nippi, Inc. K.S. is a shareholder of Matrixome, Inc.

## Data Availability

Data will be made available on request.
