# Supplementary material for: P-LM421E8, the heparan sulfate chain-conjugated laminin-421-E8 fragment, drives differentiation of human induced pluripotent stem cells into hematopoietic progenitor cells comparable to basic fibroblast growth factor in a chemically defined system
Source: Matrix Biol Plus. 2025 Dec 10;29:100188. doi: 10.1016/j.mbplus.2025.100188 (PMC12765145; doi:10.1016/j.mbplus.2025.100188)
Supplement: Supplementary Data 1 [file mmc1.pdf]

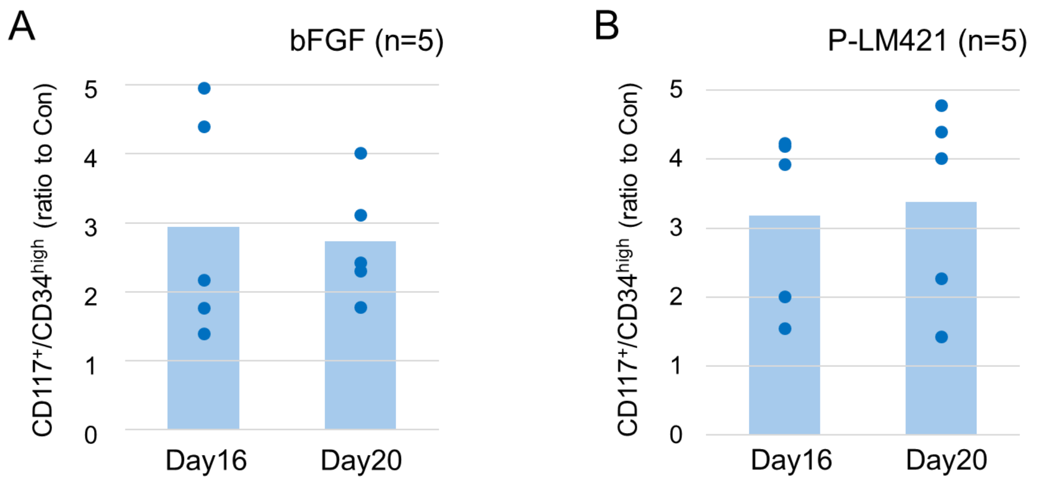

Figure S1. Effects of bFGF and P-LM421E8 on the differentiation of HPCs from hiPSCs. (A) hiPSCs were differentiated on LM511E8 with bFGF added during both the ME and HE induction periods. Floating cells collected on days 16 and 20 were analyzed by flow cytometry using anti-CD34 and anti-CD117 antibodies. Data represent the ratio of CD34-high/CD117^{+} HPCs in the presence versus absence of bFGF (Con: control) from five independent differentiation experiments. (B) hiPSCs were differentiated on LM511E8 or P-LM421E8. Floating cells collected on days 16 and 20 were analyzed by flow cytometry using anti-CD34 and anti-CD117 antibodies. Data represent the ratio of CD34-high/CD117^{+} HPCs on P-LM421E8-coated versus LM511E8-coated dishes (Con: control) from five independent differentiation experiments.

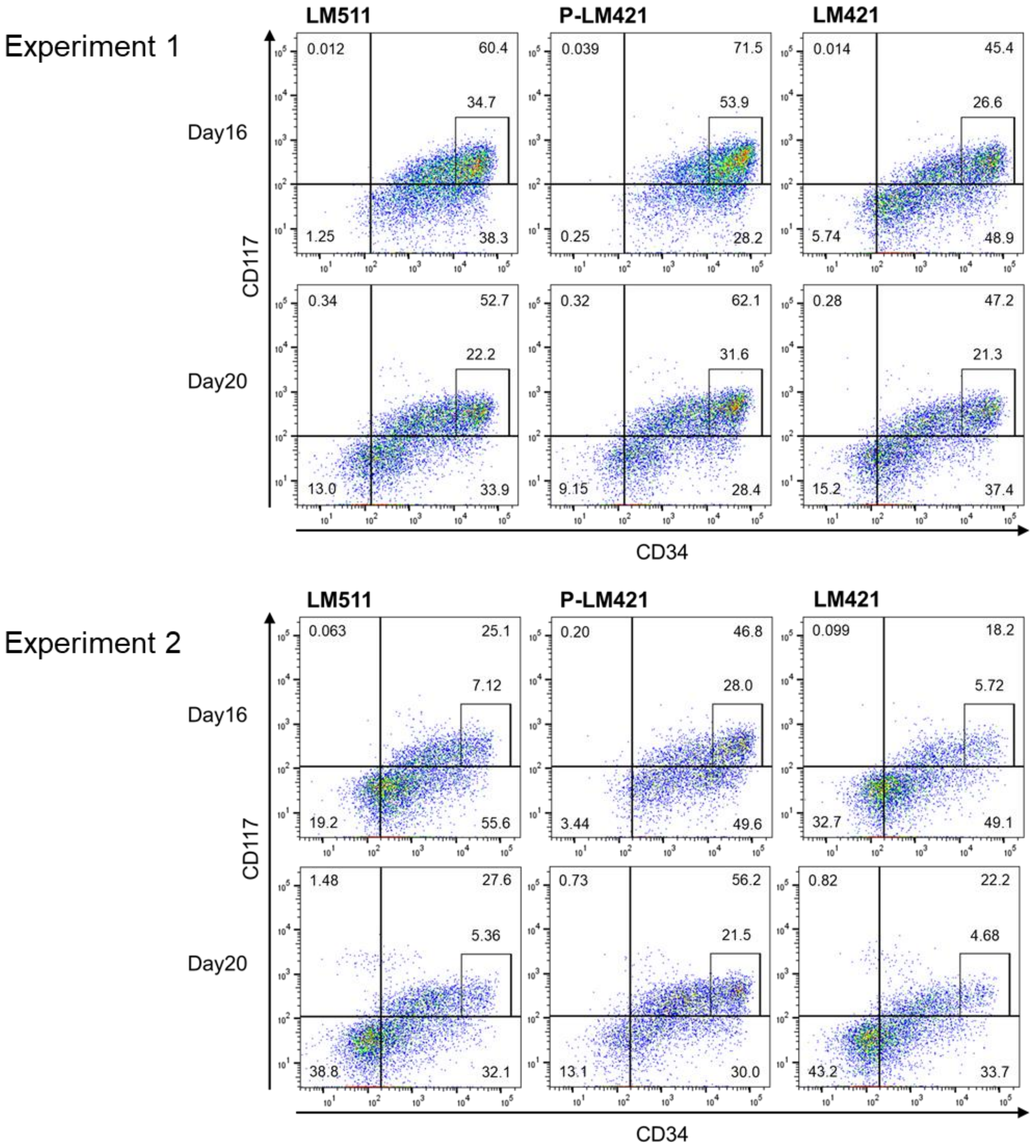

Figure S2. The role of perlecan domain 1 (D1) in P-LM421E8 on the differentiation of hiPSC-derived hematopoietic progenitor cells (HPCs).

hiPSCs were seeded at low density on LM511E8, P-LM421E8, or LM421E8 and cultured for 7 days prior to differentiation. HPCs were generated through a sequential protocol consisting of 2 days of mesoderm (ME) induction, 2 days of hemogenic endothelium (HE) induction, followed by 12 or 16 days of HPC induction. Floating cells collected on days 16 and 20 of differentiation were analyzed by flow cytometry using anti-CD34 and anti-CD117 antibodies. Boxed populations indicate CD34-high/CD117^{+} HPCs. Two independent differentiation experiments were performed (Experiment 1 and Experiment 2). The recombinant laminin fragments P-LM421E8 and LM421E8 used were produced using the FreeStyle 293 Expression System (Thermo Fisher Scientific) as described in [36]; full preparation details are provided in the Supplementary Methods.

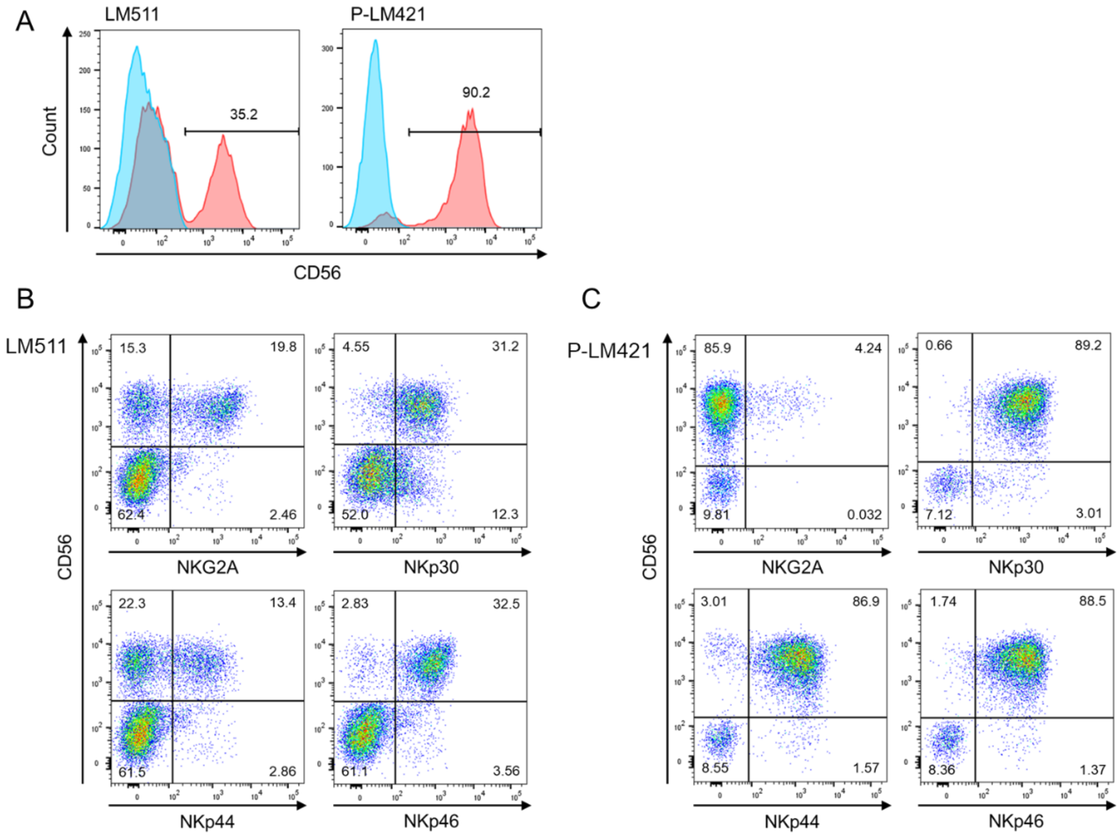

Figure S3. Comparison of NK cell differentiation using HPCs generated on LM511-E8 versus P-LM421E8.

HPCs obtained from hiPSCs seeded on LM511E8- or P-LM421E8-coated dishes were cultured under NK cell differentiation conditions for 42 days. (A) Flow cytometry was performed using anti-CD56 antibodies. Blue and red histograms represent isotype control and anti-CD56 staining, respectively. (B, C) NK cells differentiated from hiPSC-derived HPCs on LM511E8 (B) and P-LM421E8 (C) were analyzed by flow cytometry using anti-CD56 in combination with anti-NKG2A, anti-NKp30, anti-NKp44, or anti-NKp46 antibodies.

Supplementary Methods

Preparation of P-LM421E8 and LM421E8

Recombinant laminin fragments P-LM421E8 and LM421E8 were produced using expression vectors encoding the α4-E8 fragment either fused at its C-terminus to perlecan domain 1 (D1) (P-LM421E8) or without D1 (LM421E8), together with β2-E8 and γ1-E8. The α4-E8 (±D1), β2-E8, and γ1-E8 fragments were N-terminally tagged with 6×His, HA, and FLAG, respectively. Proteins were expressed using the FreeStyle 293 Expression System (Thermo Fisher Scientific) and purified from conditioned media by sequential chromatography on Ni-NTA agarose (Qiagen) and anti-FLAG M2 agarose (Sigma). During purification of P-LM421E8, the Ni-NTA wash step included TBS (without Ca^{2+}/Mg^{2+}) containing 1 M NaCl to remove materials bound via heparan sulfate chains on D1. Purified P-LM421E8 and LM421E8 were dialyzed against PBS (without Ca^{2+}/Mg^{2+}), pH 7.4. Protein concentrations were determined using a BCA protein assay kit (Thermo Fisher Scientific) with bovine serum albumin as the standard. iMatrix-511 (Matrixome) was used as the LM511E8 substrate. Coating was performed at 1.0 μg/cm^2 for P-LM421E8 or LM421E8 and 0.5 μg/cm^2 for LM511E8 in PBS (without Ca^{2+}/Mg^{2+}), pH 7.4.
